# Supplementary figures and images for: Single-domain antibody inhibitors target the coiled coil arms of the Bacillus subtilis SMC complex
Source: eLife. 2026 May 19;15:RP111131. doi: 10.7554/eLife.111131 (PMC13186564; doi:10.7554/eLife.111131)

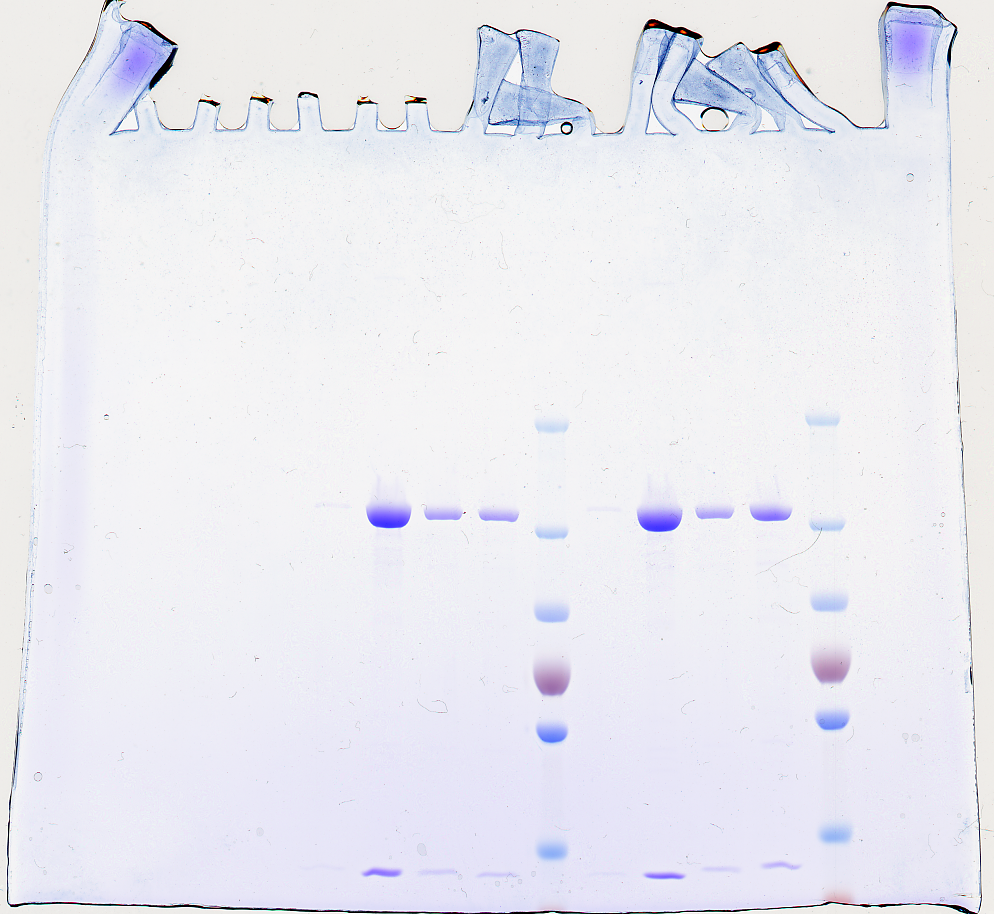

Supplement: Figure 1—figure supplement 1—source data 1. [file elife-111131-fig1-figsupp1-data1.zip › Source data 1.tif]

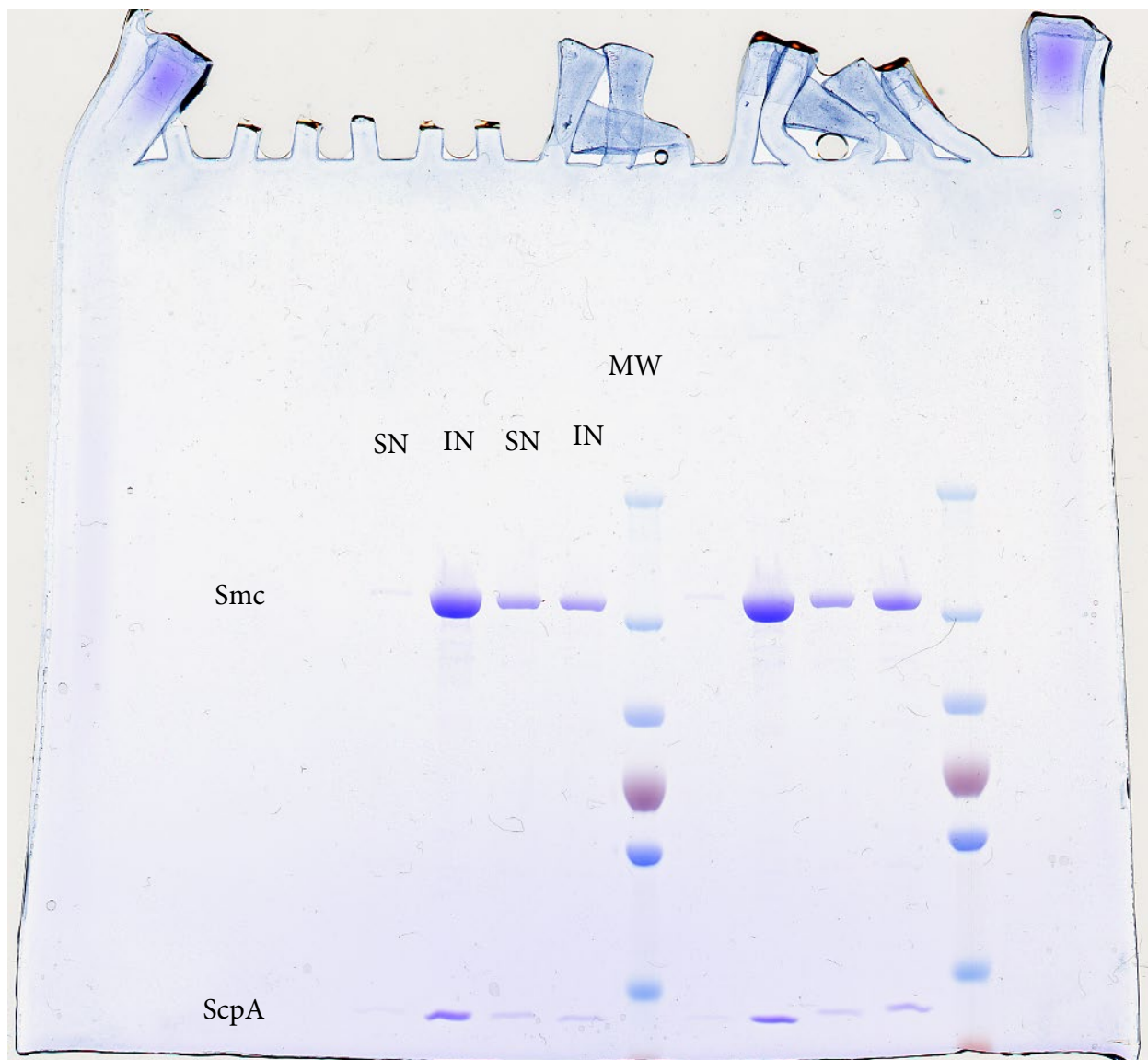

Supplement: Figure 1—figure supplement 1—source data 2. [file elife-111131-fig1-figsupp1-data2.zip › Source data 2.pdf]
